# Looking beyond the imaging plane: 3D needle tracking with a linear array ultrasound probe

**DOI:** 10.1038/s41598-017-03886-4

**Published:** 2017-06-16

**Authors:** Wenfeng Xia, Simeon J. West, Malcolm C. Finlay, Jean-Martial Mari, Sebastien Ourselin, Anna L. David, Adrien E. Desjardins

**Affiliations:** 1 Department of Medical Physics and Biomedical Engineering, University College London, Gower Street, London, WC1E 6BT United Kingdom; 2 Wellcome/EPSRC Centre for Surgical and Interventional Sciences, University College London, Charles Bell House, 67-73 Riding House Street, London, W1W 7EJ United Kingdom; 3 Department of Anaesthesia, University College Hospital, Main Theatres, Maple Bridge Link Corridor, Podium 3, 235 Euston Road, London, NW1 2BU United Kingdom; 4 St Bartholomew’s Hospital and Queen Mary University of London, Charterhouse Square, London, EC1M 6BQ United Kingdom; 5 GePaSud, University of French Polynesia, Faa’a, 98702 French Polynesia; 6 Centre for Medical Imaging Computing, University College London, Gower Street, London, WC1E 6BT United Kingdom; 7 Institute for Women’s Health, University College London, 86-96 Chenies Mews, London, WC1E 6HX United Kingdom

## Abstract

Ultrasound is well suited for guiding many minimally invasive procedures, but its use is often precluded by the poor visibility of medical devices. When devices are not visible, they can damage critical structures, with life-threatening complications. Here, we developed the first ultrasound probe that comprises both focused and unfocused transducer elements to provide both 2D B-mode ultrasound imaging and 3D ultrasonic needle tracking. A fibre-optic hydrophone was integrated into a needle to receive Golay-coded transmissions from the probe and these data were processed to obtain tracking images of the needle tip. The measured tracking accuracy in water was better than 0.4 mm in all dimensions. To demonstrate the clinical potential of this system, insertions were performed into the spine and the uterine cavity, in swine and pregnant ovine models *in vivo*. In both models, the SNR ranged from 13 to 38 at depths of 22 to 38 mm, at out-of-plane distances of 1 to 15 mm, and at insertion angles of 33 to 42 degrees relative to the probe surface normal. This novel ultrasound imaging/tracking probe has strong potential to improve procedural outcomes by providing 3D needle tip locations that are co-registered to ultrasound images, while maintaining compatibility with current clinical workflow.

## Introduction

A long standing problem in minimally invasive procedures guided with ultrasound (US) imaging is the identification of the medical device tip within the body. This problem is experienced acutely during percutaneous interventions in many clinical fields, including regional anaesthesia and interventional pain management^1^, interventional oncology^2^, and foetal medicine^3^. Ultrasonic visualisation of medical devices such as needles can be very challenging when the distal ends are not coincident with the US imaging plane. This occurs frequently in clinical practice: during “in-plane” insertions, a thin needle can readily stray from the imaging plane; during “out-of-plane” insertions, only a small region of the needle in the vicinity of the imaging plane can be visualised, and in this region, the needle tip can have a very similar appearance to that of the needle shaft. Additionally, medical device visibility can be lost during steep, large-angle insertions when US waves are reflected away from the US probes^4^. Serious complications can arise from loss of visibility of the medical device. For instance, inadvertent injections of particulate steroids into a blood vessel during interventional pain management procedures can result in stroke^5^.

A number of solutions have been proposed to improve medical device visibility during US-guided procedures, but their limitations have precluded widespread clinical use. Echogenic surfaces can be useful for large angle insertions during in-plane insertions. However, they are not relevant to visualizing medical devices outside the US imaging plane^6^. Electromagnetic (EM) tracking can provide out-of-plane tracking, but its accuracy can be severely degraded by EM field disturbances such as those arising from metal in operating theatre tables^7^, and the sensors integrated into needles tend to be expensive. US imaging in 3D can readily visualise medical devices when they are surrounded by fluid, for instance in certain cardiac and foetal procedures^8^. However, US imaging in 2D is preferred for most minimally invasive procedures due to simplicity of image interpretation; current US probes that provide 3D imaging tend to have insufficient resolution for frequently-performed peripheral applications such as nerve blocks and central venous cannulations.

Ultrasonic tracking is a solution that has recently been the focus of intense development^9–13^. With this method, there is ultrasonic communication between the external US probe and a transducer integrated within the medical device. The medical device can be localised from measurements of the transmission times between the transducer and different elements of the probe. To bring ultrasonic tracking into a broad range of clinical applications, it is crucial to provide out-of-plane localisation of the medical device using compact probes for 2D imaging. To date, meeting this challenge has proven elusive: ultrasonic tracking with conventional probes for 2D imaging only localises the distal ends of medical devices when they are within the imaging plane.

Here, we demonstrate that 3D tracking can be performed with a US probe for 2D imaging that is applicable in a wide range of clinical contexts. Reception of US imaging transmissions was performed with a fibre-optic hydrophone (FOH) sensor integrated into a spinal needle. Clinical utility was tested using *in vivo* swine and pregnant sheep models.

## Results

### 3D ultrasonic tracking

**Figure 1:**
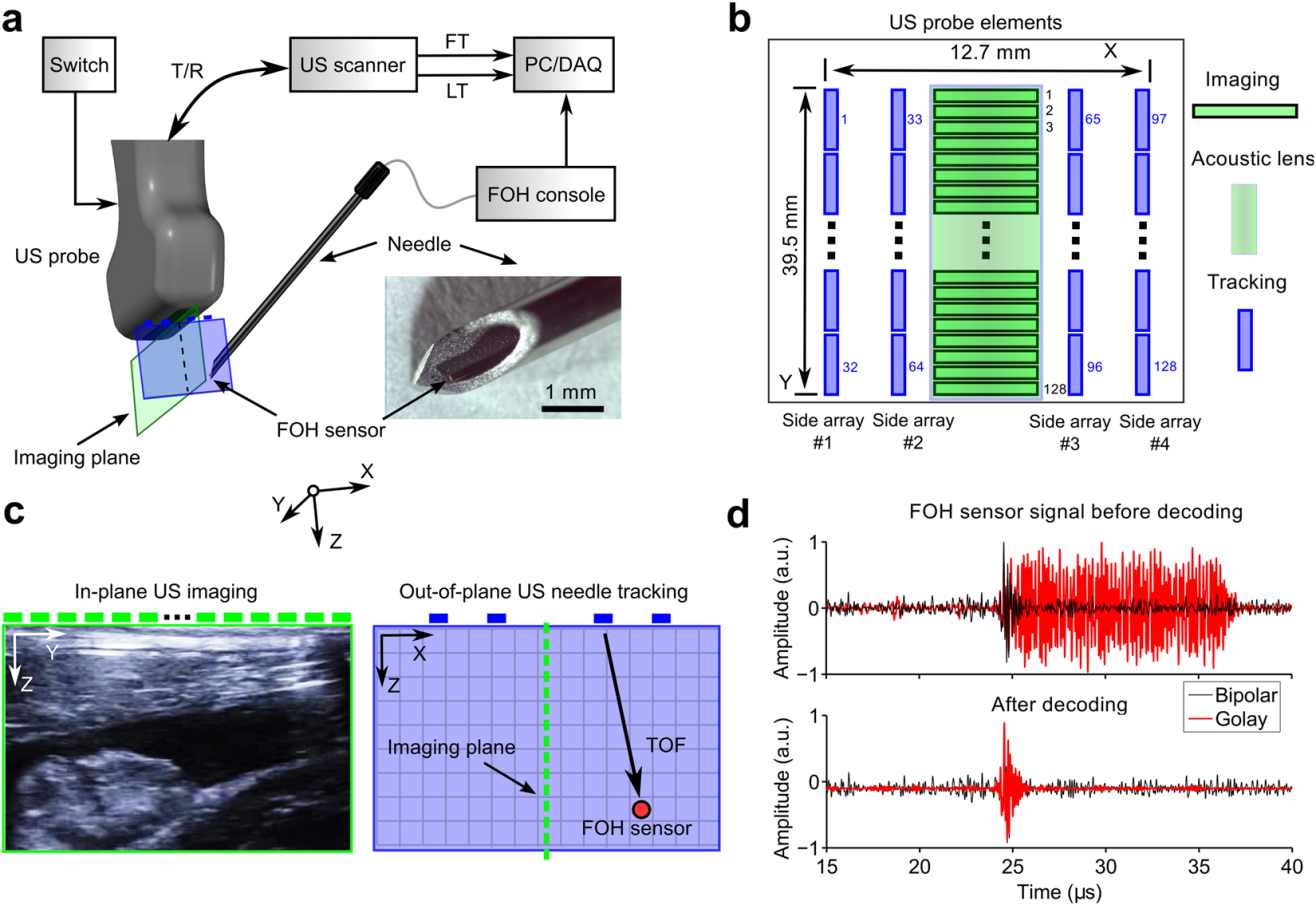
System overview. (**a**) Schematic showing the ultrasound (US) imaging/tracking probe developed in this study that allows for 2D US imaging and 3D needle tracking. A switch allowed for rapid electronic selection of transducer elements for imaging or those for tracking. The probe was driven by a commercial US scanner that allowed for control over transmissions from individual tracking elements. A fibre optic hydrophone (FOH) sensor, positioned within the lumen of a 20 gauge needle, received tracking transmissions. T/R: transmit/receive; LT: line trigger; FT: frame trigger; PC: personal computer; DAQ: digital acquisition card. (**b**) Transducer element layout of the probe: a focused central array was used for imaging (128 elements); side arrays, for tracking (32 elements per row; 128 elements in total). (**c**) Information derived from the transducer arrays: a sample B-mode US image acquired with the central array; a transmission from a side array element received by the FOH sensor is shown schematically. TOF: time-of-flight. (**d**) Coded excitation scheme: raw signals received by the FOH sensor corresponding to a Golay transmission and a bipolar pulse (top); decoded signal from a Golay transmission pair and a raw bipolar pulse are shown for comparison (bottom).

To achieve 3D needle tracking with a clinical US probe for 2D imaging, we developed a custom US tracking/imaging probe and integrated a miniature FOH sensor into the needle (Fig. 1a). This custom probe had two sets of transducer elements for imaging and tracking, respectively (Fig. 1b). The central, 1D array of 128 elements for B-mode imaging and the associated cylindrical lens were similar to those of 9–4 MHz linear array probes used in current practice. On each side of this array, there were two 32-element side-arrays for tracking that were unfocused, so that their transmissions extended outside the imaging plane (Fig. 1c). The probe had a single connector that was compatible with a clinical US imaging system for 2D imaging. A switch within the tracking/imaging probe allowed this connector to access either the central array or the 4 side arrays. When in tracking mode, transmissions from each element were performed sequentially, starting with the first side array and progressing to the fourth. Ultrasound transmissions from tracking elements were received by a fibre-optic hydrophone (FOH) sensor^14^ that was positioned unobtrusively within the cannula, so that it did not protrude beyond the bevel surface. The small diameter of the FOH sensor allowed for concurrent US reception and fluid injections.

Golay coding^15^ of the US transmissions used for tracking was performed to increase the signal-to-noise ratio (SNR) of signals received by the FOH sensor. As compared with short bipolar transmissions that are typically used for US imaging, coded transmissions were extended in time. With pulse compression applied to Golay coded transmissions, the temporal profile of the FOH sensor signal received with bipolar transmissions was recovered (Fig. 1d).

**Figure 2:**
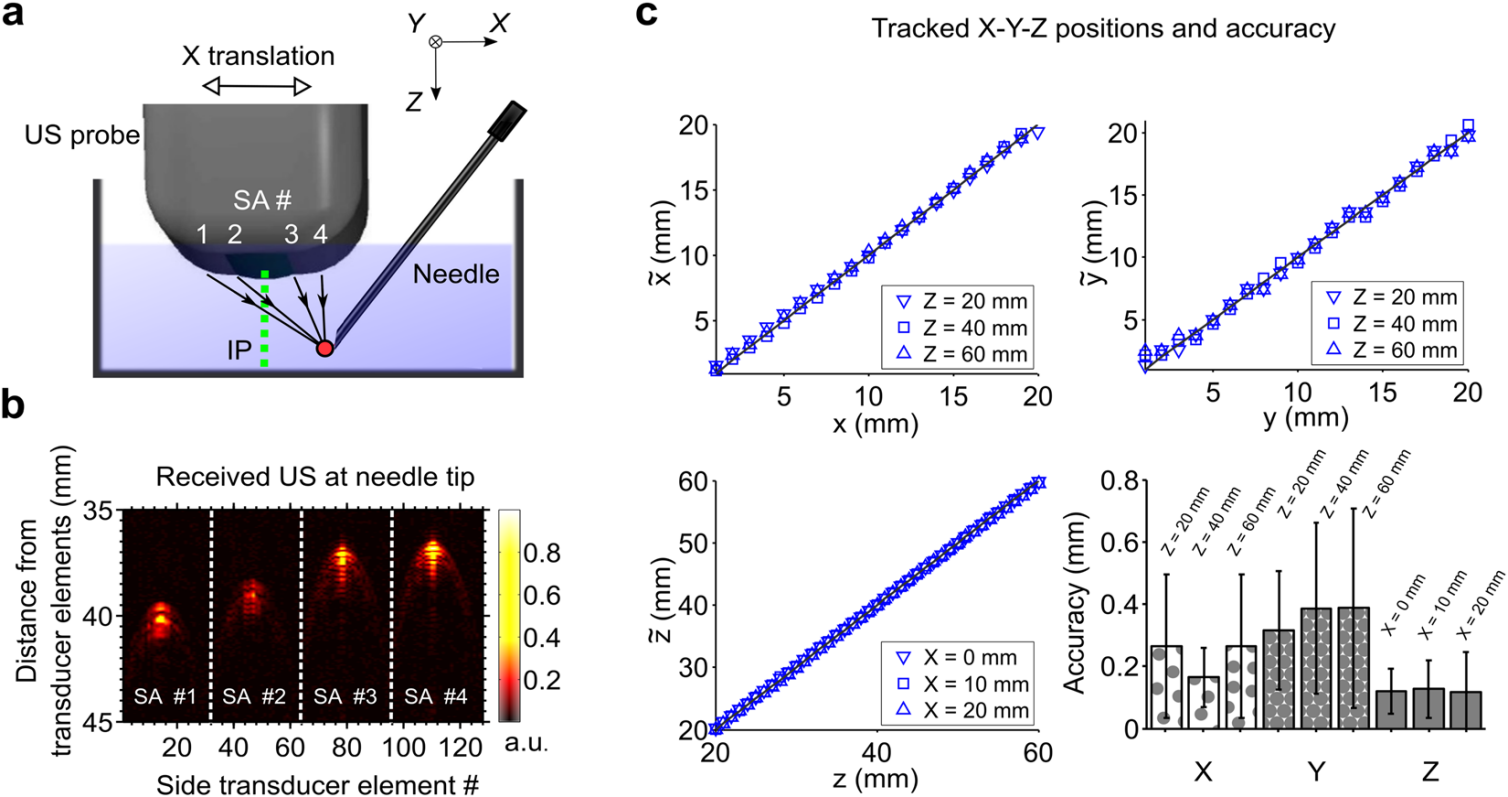
3D tracking accuracy measurements. (**a**) With the needle tip and imaging/tracking probe in a water bath, transmissions from tracking transducer elements in the 4 side arrays (SA) of the probe were received by the fibre optic hydrophone (FOH) sensor at the tip of needle (red circle). The needle was fixed in position and angle, and the probe was translated in 3 orthogonal directions (translation in X is shown). IP: imaging plane (dashed green line). (**b**) Decoded Golay-coded signals received by each tracking element of the probe were concatenated and displayed on a linear scale (expanded vertical axis). The central maximum observed in the decoded signals from each side array corresponded to reception of transmissions from the hydrophone. (**c**) Tracked $(\tilde{x},\tilde{y},\tilde{z})$ and actual $(x,y,z)$ positions, obtained from translations in X (top left), Y (top right), and Z (bottom left), were in close agreement. Translations in the X and Y directions were performed at 3 needle tip depths; likewise, translations in the Z direction were performed at 3 different out-of-plane distances. Compiled accuracy statistics are plotted as mean ± standard deviation (bottom right).

With the needle in water, pulse-compressed transmissions from all four side arrays (Fig. 2a) were apparent at depths up to 6 cm and out-of-plane distances up to 2 cm. When the FOH sensor signals were concatenated to form four images corresponding to the four side arrays, a single region of high signal values was observed in each of these images (Fig. 2b). The spatial locations of these high-signal regions were processed to obtain the needle tip location (Methods).

### Tracking accuracy: measurements in water

The spatial locations of the tracked and actual needle positions in water had excellent agreement for all needle tip positions (Fig. 2c). Across all 240 measurement points considered in this study, which included depths (Z) in the range of 20 to 60 mm, out-of-plane distances (X) from 0 to 20 mm, and all lateral distances (Y) spanned by the transducer elements, the mean spatial accuracy was finer than 0.38 mm. Overall, accuracy was finest in the Z dimension and lowest in the Y dimension. In the X dimension, the accuracy varied with Z; it was finest in the vicinity of 40 mm (accuracy: 0.16 mm).

### Tracking accuracy: simulations with speed of sound heterogeneity

**Figure 3:**
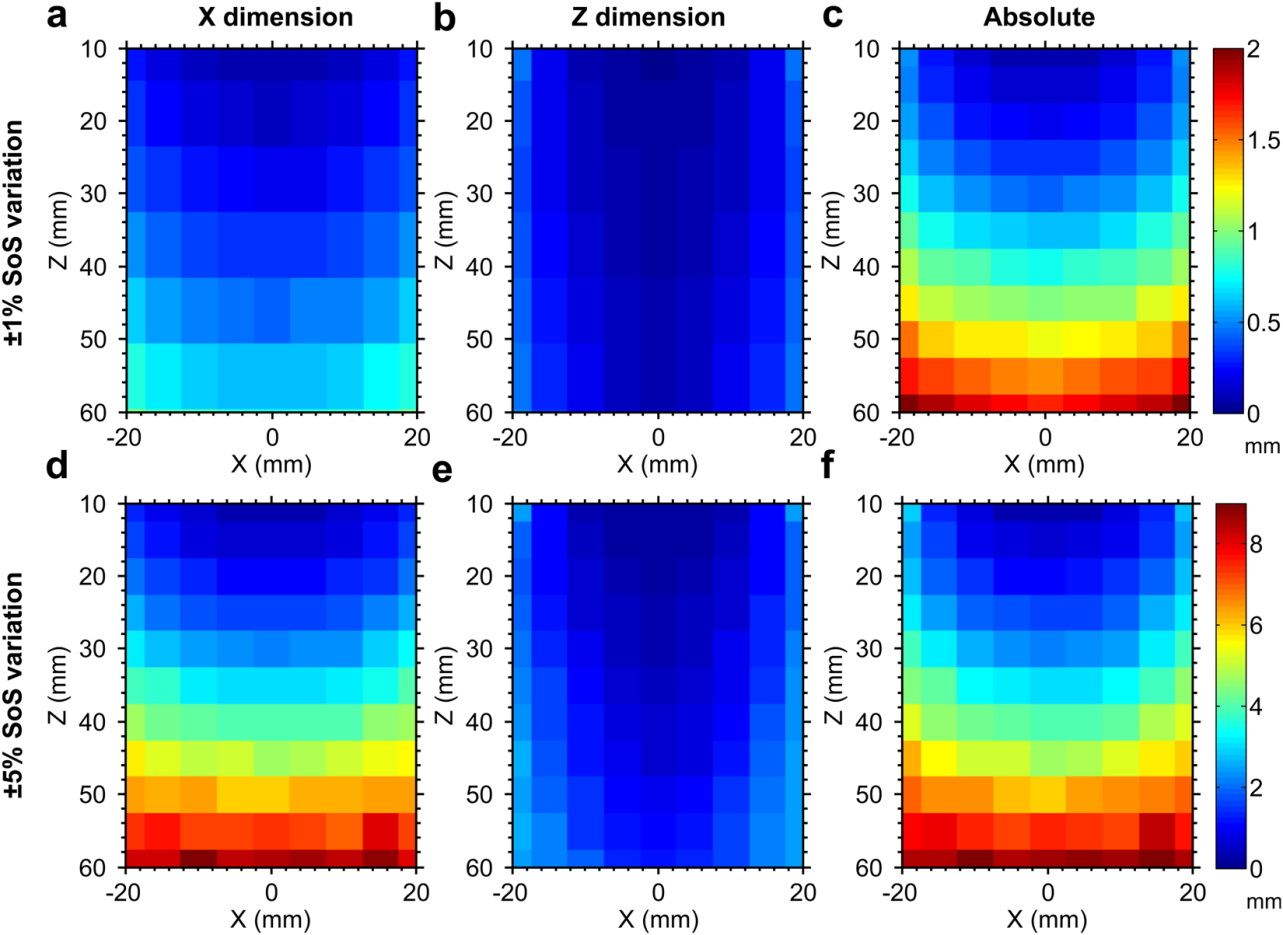
Effects of tissue speed of sound (SoS) heterogeneity on tracking accuracy with Monte Carlo simulation. The SoS in tissue varied randomly with a variation of 1% (**a–c**) or 5% (**d–f**) in these simulations, whilst the assumed SoS used for obtaining tracking estimates was held constant at 1540 m/s. Tracking accuracy in the Z (depth) dimension (**b**,**e**) was better than that in the X (out-of-plane) dimension (**a**,**d**). The absolute tracking accuracy (**c**,**f**) tended to improve with decreasing depth and with decreasing out-of-plane distance.

In a simulation study in which the speed of sound (SoS) in tissue varied randomly whilst the assumed SoS used for obtaining tracking estimates was held constant, the tracking accuracy varied across the Z (depth) and X (out-of-plane) dimensions, and with the magnitude of the SoS variations (Fig. 3). In general, tracking accuracy in Z (Fig. 3b,e) was finer than that in X (Fig. 3a,d). The absolute tracking accuracy tended to worsen with increasing depth and with increasing out-of-plane distance (Fig. 3c,f). At the depth limit of the simulations (60 mm), some counterexamples to this trend were observed, which are likely due to the limited number of Monte Carlo iterations. For a SoS variation of 1%, the mean absolute tracking accuracy across the spatial grid was 0.85 mm; it attained a maximum of 2.06 mm at Z = 60 mm and X = −20 mm. For a SoS variation of 5%, the mean and maximum of the absolute tracking accuracy were 4.16 mm and 9.29 mm, respectively, with the latter attained at Z = 60 mm and X = −10 mm.

### Validation in a swine model *in vivo*

**Figure 4:**
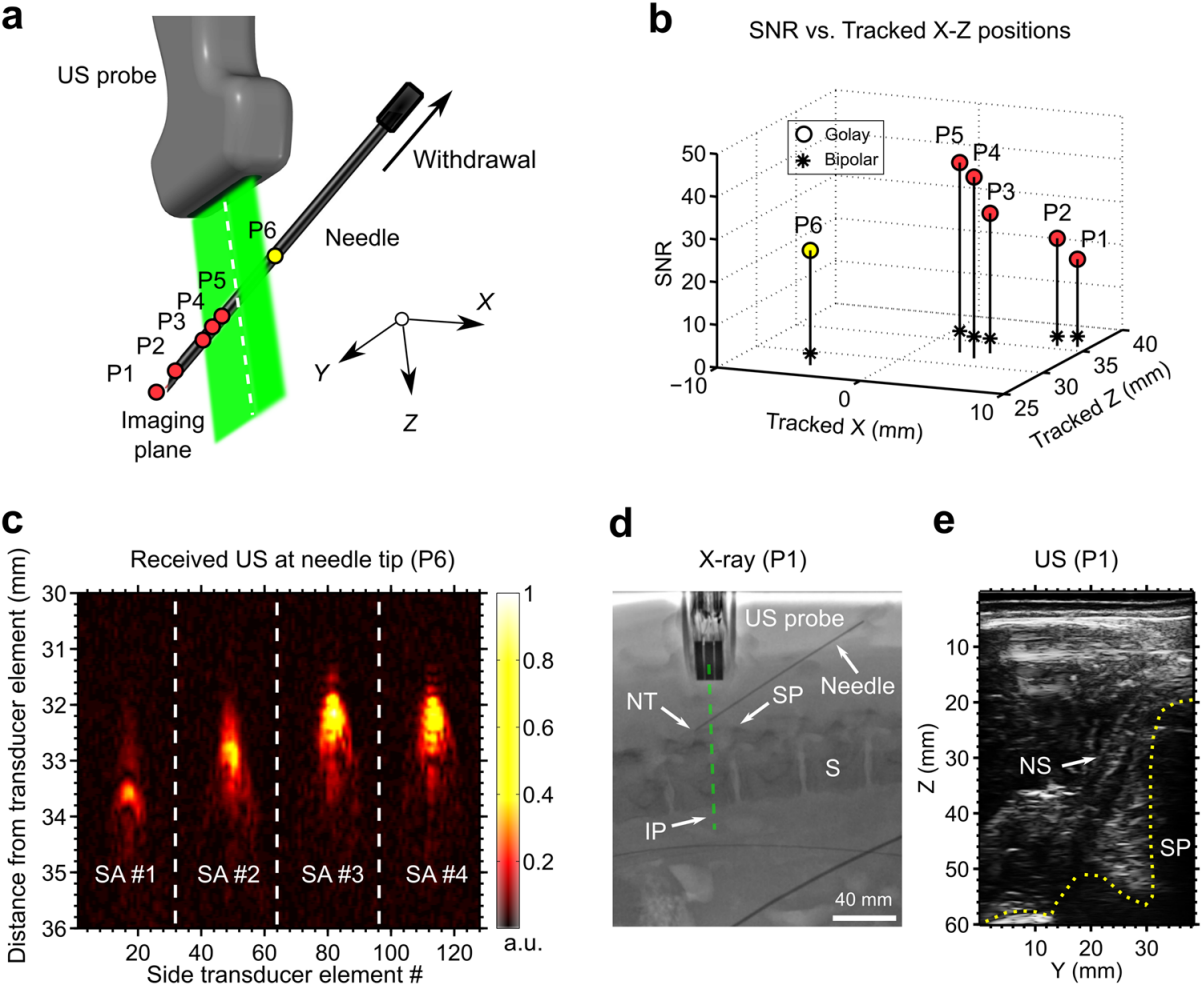
Needle insertion into the spinal region of a swine *in vivo* with concurrent 3D tracking. (**a**) Tracked positions (circles: P1–P6) obtained during withdrawal of the needle; the circle colour (red/yellow) indicates the side of the ultrasound (US) imaging plane in which the needle tip resided. The imaging plane is depicted in green; the white dashed line indicates intersection between the imaging plane and the lateral position of the needle shaft. (**b**) The signal-to-noise ratio (SNR) of the decoded US signals at the needle tip increased with proximity to the imaging plane. (**c**) In the concatenated decoded signals received from tracking transducer elements of the 4 side-arrays (SA), prominent maxima were observed (expanded vertical scale). These maxima corresponded to transmissions received by the needle tip (signals correspond to tracked position P6). (**d**) Out-of-plane needle tip placement was confirmed with X-ray fluoroscopy (image corresponds to tracked position P1). NT: needle tip; SP: spinous process; S: spine; IP: imaging plane. (**e**) With US images acquired from the central array of transducer elements, the needle shaft was faintly visible. The apparent location of the needle shaft on US imaging was consistent with the tracked positions, extrapolated to a zero out-of-plane distance (image corresponds to tracked position P1). NS: needle shaft; SP: spinous process.

An out-of-plane insertion performed on the lumbar region of a swine *in vivo* provided a preliminary indication of the system’s potential for guiding regional anaesthesia and interventional pain management. The needle tip was tracked at multiple locations during manual withdrawal, on both sides of the imaging plane (Fig. 4a). According to these tracking data, the needle tip reached an out-of-plane distance greater than 9 mm and a depth greater than 35 mm. For all positions, SNRs greater than 20 were obtained with Golay coding, with a mean value of 32. This mean SNR was 9.1 times larger than that obtained with conventional bipolar excitation (Fig. 4b). These high SNRs stemmed from clear images of the needle tip acquired from the side arrays, in which there were unique and prominent maxima (Fig. 4c). The SNR increased with proximity of the needle tip to the US imaging plane (Fig. 4b). X-ray fluoroscopy provided confirmation that the needle tip was out-of-plane at the deepest point of the insertion (Fig. 4d). With transverse US imaging performed with the central array of transducer elements, the needle shaft was faintly visible when it crossed the imaging plane; its visibility manifested as slight tissue deformations during insertion. The region in the US image in which the needle shaft resided was consistent with the tracked needle tip positions extrapolated to the imaging plane (Fig. 4e).

### Validation in a sheep model *in vivo*

**Figure 5:**
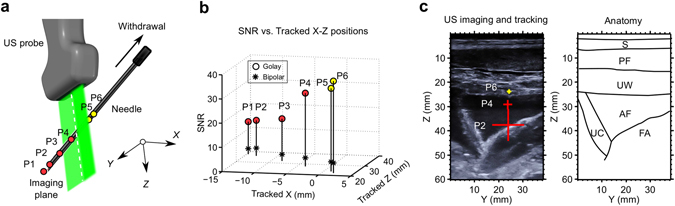
Needle insertion into the uterine cavity of a pregnant sheep *in vivo* with concurrent 3D tracking. (**a**) Tracked needle tip positions (circles: P1-P6) obtained during withdrawal of the needle, displayed as they are in Fig. 4. (**b**) The signal-to-noise ratio (SNR) of the decoded US signals at the needle tip tended to increase with proximity to the imaging plane. (**c**) The tracked positions were overlaid with crosses onto a 2D US image (left) acquired with the central array of transducer elements, with the size of the cross (end-to-end of each line) scaled to represent the out-of-plane distance (for clarity, only 3 positions out of 6 are shown). The crosses were coloured (red/yellow) according to the side of the imaging plane in which the needle tip resided. Key anatomical features are depicted with outlines (right). S: skin; PF: percutaneous fat; UW: uterus wall; AF: amniotic fluid; UC: umbilical cord; FA: foetal abdomen.

With a needle insertion performed into the uterine cavity of a pregnant sheep *in vivo*, the needle tip was tracked at out-of-plane distances up to 15 mm and depths up to 38 mm (Fig. 5a). Despite the presence of physiological tissue motion similar to that encountered in clinical practice, SNRs greater than 13 were obtained with Golay coding at all locations, with a mean value of 25. This mean SNR was 7.5 times larger than that obtained with conventional bipolar excitation (Fig. 5b). As observed in the swine model, the SNR tended to increase with the proximity of the needle tip to the imaging plane. The 3D tracking information was overlaid on the 2D US image (Fig. 5c). With this overlay, the lateral and depth coordinates of the needle tip were indicated with the centre of a cross; the out-of-plane distance was indicated with the size of this cross (length of each line). The cross was coloured according to the side of the imaging plane in which the needle tip resided (Fig. 5c).

## Discussion

We have shown that clinical linear-array US probes that are widely used to guide minimally invasive procedures can be modified in a simple manner to provide high resolution 3D tracking of medical devices. Several aspects of the US imaging/tracking probe developed in this study are attractive from the standpoint of clinical translation. First, its dimensions are similar to those of conventional linear array probes used for 2D US imaging, so that it can be used in small spaces such as the axilla where manoeuvring bulky probes for 3D US imaging is challenging. Second, the US imaging/tracking probe is compatible with commonplace 2D US imaging systems such as those used for point-of-care applications: with an electronic switch to alternate between imaging and tracking elements, a standard connector block used for relaying US signal transmissions to the US console can be used. Tracking in 3D can be achieved without compromising US image quality or sufficiently increasing the complexity of the probe and its interactions with the US imaging console.

The FOH sensor is ideally suited to ultrasonic needle tracking. With its small diameter and flexibility, it can readily be integrated into devices with small lateral dimensions. Integration into needles in a manner that allows for concurrent fluid injections, as demonstrated in this study, is important for peripheral nerve blocks where injections can be performed at multiple locations during one insertion. Its wide bandwidth^14^ allows for compatibility with many different US probes, and it could allow for tracking with transducer elements of different frequencies within one probe. Additionally, its near-omnidirectional response^14^ is useful to allow for tracking across a wide range of needle insertion angles. In contrast to EM sensors, the FOH sensor is immune to interference from metal objects and EM fields. To increase the range of depths at which sensing can be performed, FOH sensors with a plano-concave cavity that have greater US sensitivity could be used^16^. Ultimately, the same fibre optic used for US sensing could also be used for optical sensing of the tissue environment with modalities such as reflectance spectroscopy^17–22^, Raman spectroscopy^23^, optical coherence tomography^24, 25^, and photoacoustic imaging^26–28^.

The time required to perform US tracking transmissions is sufficiently small to allow for 2D B-mode US imaging at video rates. For instance, with tracking and imaging arrays that each comprise 128 elements, and with an imaging depth of 10 cm, pairs of tracking and B-mode US image frames could be acquired at 30 Hz. Beyond this scheme, there is significant scope for reducing the time required to perform US tracking. For instance, transmissions from tracking transducer elements could be performed with orthogonal codes, so that they could be performed simultaneously and subsequently decoded from a single FOH signal. With the current system, tracking images were acquired and saved at 1 to 2 Hz to allow for sufficient time to transfer data from the digital acquisition card, which included both bipolar and Golay coded tracking transmissions.

Challenges with identifying the needle tip that are routinely encountered during US-guided interventions in current clinical practice can be overcome with ultrasonic tracking. Here, tracking images provide uncluttered views of the needle tip in which only this object is present. This is particularly important in heterogeneous tissue environments such as those encountered in peripheral nerve blocks, in which there are often linear features that can be readily be misinterpreted as needles^29^. Given that US tracking involves only one-way transmission between the US probe and the needle, the needle tip can be tracked even when its shaft is sufficiently angled to be invisible on B-mode US images. As demonstrated in this study, needle trajectories can be inferred from a sequence of tracked needle tip positions obtained with a single FOH sensor. In future implementations, multiple FOH sensors could allow for an estimate of the needle trajectory from a single position.

The sub-mm tracking accuracy obtained from measurements in water is likely to be sufficient for most US-guided interventional procedures, as it is smaller than the inner diameter of 20 gauge needles. In this study, a numerical approach was used to estimate the needle tip position, in which the differences between simulated and measured time-of-flights were minimised to obtain a needle position estimate. In future implementations, the minimum difference could be used as a confidence measure of the estimate and displayed alongside the tracked needle positions. As an alternative to the numerical approach used here, analytical close-form approaches such as the spherical interpolation method^30^ could be used.

With ultrasonic tracking, the impact of heterogeneities of the tissue acoustic properties depends on how tracking is performed. With the US imaging/tracking probe presented here, spatial variations in the SoS of tissue will distort the apparent position of the needle tip, most prominently in the out-of-plane dimension, as demonstrated by the simulation study. Incorporation of anatomical information and corresponding variations in the SoS will improve tracking accuracy. When the needle tip is in the imaging plane, tracking data from the central array of transducer elements, which are naturally co-registered to the US images^12, 13^, could supplement tracking data from the side arrays. Tracking data from previous needle positions could be used to estimate the needle tip trajectory. Requirements for tracking accuracy in the depth and out-of-plane dimensions are likely to depend on the clinical procedure being performed, and on the current depth and proximity to the image plane of the needle tip. With in-plane insertions, the primary role of out-of-plane tracking may be to determine on which side of the imaging plane the needle tip resides, in order to reorient the imaging probe to bring the needle in-plane. As such, enabling this role may be more important than achieving high tracking accuracy in the out-of-plane dimension. Ultrasonic tracking at locations directly behind bony structures or air cavities is likely not possible, in the same way that these regions are typically not visualised on US imaging. In future experiments, position estimates obtained with 3D rotational C-arm computed X-ray tomography could be used to assess the accuracy of 3D ultrasonic tracking in heterogeneous tissues *in vivo*.

Despite recent improvements in US image quality, interpretation of images with this modality and coordinated manipulated of medical devices remains challenging, even for expert practitioners. Increased interactions between US probes and medical devices will improve procedural safety and efficiency, and they could facilitate clinical training by providing needle trajectories^31^. In several clinical contexts, these improvements could greatly facilitate adoption of US in place of X-ray fluoroscopy, for instance in spinal insertions for interventional pain management where out-of-plane insertions are often required. Ultrasonic 3D tracking with compact US probes for 2D imaging has strong potential to improve procedural outcomes by providing full awareness of the needle tip location, while maintaining compatibility with current clinical workflow.

## Methods

### System

The custom US imaging/tracking probe (Vermon, Tours, France) comprised five rows of transducer elements to perform 3D tracking and 2D B-mode US imaging. It was driven by a commercial US imaging system (SonixMDP, Analogic Ultrasound, Richmond, BC, Canada), operated in research mode (Fig. 1a). The central row of 128 imaging elements was similar to that of a linear 9–4 MHz imaging US probe. On each side of this array, there were two 32-element side-arrays for tracking that were unfocused. The elements in both the central and side arrays had a nominal bandwidth of 4–9 MHz. The transducer elements in the central array (0.3 mm × 4 mm) were oriented so that their longer sides were adjacent to each other, which is a typical arrangement for a linear array US imaging probe. Those in the side arrays (0.3 mm × 1.2 mm) had the opposite orientation (Fig. 1b). This orientation of the side array elements was chosen to maximise the spatial coverage of US transmissions beyond the imaging plane. The central imaging row was fitted with a cylindrical acoustic lens for out-of-plane focusing. The side tracking rows were unfocused, for out-of-plane transmissions.

Transmissions from the US imaging/tracking probe were received by a FOH sensor within a 20 gauge spinal needle (inner diameter: 0.6 mm; Terumo, Surrey, UK). The sensing region of the FOH was a Fabry-Pérot cavity (outer diameter: 150 µm) at the distal end of a single-mode optical fibre. This cavity, which comprised a parylene spacer between two gold reflective layers (Precision Acoustics, Dorchester, UK), was interrogated by a wavelength-tuneable laser^14^. The FOH sensor console provided a signal proportional to the time-varying reflectivity of the cavity with a wavelength-tuneable laser. With the wavelength tuned to optimise sensitivity of the cavity to impinging US waves, the reflectivity signal was proportional to the US pressure.

Three US transmission sequences were used for tracking, and one was used for B-mode US imaging. The first tracking sequence comprised bipolar pulses; the second and third were 32-bit Golay code pairs generated from bipolar pulses^15^. Tracking sequences were performed from individual transducer elements, sequentially across rows (Fig. 1b). All bipolar pulses used for tracking had a central frequency of 4 MHz. B-mode sequences were performed with bipolar pulses (9 MHz) from an aperture of 64 elements and an electronic focusing depth of 30 mm.

Acquisition FOH sensor data was synchronised with US transmissions using two trigger signals: a frame trigger (FT) for the start of all 128 transmissions, and a line trigger (LT) for each transmission. For each FT, FOH signals and the LT were digitised at 100 MS/s (USB-5133, National Instruments, Austin, USA)^12, 13^. The US imaging/tracking probe was controlled by a custom program operating on the US imaging system written in LabVIEW (National Instruments, Austin, USA).

### Coded excitation

Coded excitation of the US transmissions was performed with a 32-bit Golay code pair to increase the SNRs of the FOH signals. This scheme is described in detail in ref. 13. Encoding was performed by convolving a bipolar base transmission sequence with each Golay code pair sequence; the output was used to excite individual transducer elements for US transmissions. The received US signals from the FOH were band-pass filtered (Chebyshev Type I; 5th order; 2–6 MHz). Decoding was performed with two steps. First, the filtered FOH sensor data was convolved with each of the time-reversed versions of the oversampled Golay code pair sequences to generate two decoded sequences. Second, these two decoded sequences were summed, which mitigated the range side lobes^13^. The summed data were concatenated to form 4 tracking images, with each image corresponding to transmissions from one side array.

### Tracking algorithms

An estimate of the needle tip position ($\tilde{x}$, $\tilde{y},$ $\tilde{z}$) in the coordinate space of the US imaging/tracking probe was obtained from the 4 tracking images. In each of these images, the horizontal and vertical coordinates were the transducer element number and the distance from the corresponding transducer element, respectively. For the *k* ^th^ image (*k* = {1, 2, 3, 4}), the coordinate at which the signal was maximal was denoted as (*h* ^(*k*)^, *v* ^(*k*)^). Typically, the *h* ^(*k*)^ values were consistent across the 4 tracking images (Fig. 2b), so that their mean, offset from centre and scaled by the distance between transducer elements, was used to obtain the estimate $\tilde{y}$. The first step to obtain the estimates $\tilde{x}$ and $\tilde{z}$ was to calculate the time-of-flights *t*_*m*_^(*k*)^ = *v* ^(*k*)^/*c*, where *c* is the SoS. For measurements in water, *c* was calculated based on the measured water temperature^32^; for measurements *in vivo*, a standard clinical *c* value of 1540 m/s was used. The second step was to compare these *t*_*m*_^(*k*)^ values with a set of simulated time-of-flight values ${t}_{s}^{(k)}({x}_{i},\,{z}_{j})$, which were pre-computed at each point (*x*_*i*_, *z*_*j*_) of a 2D grid in the X-Z coordinate space of the US imaging/tracking probe that was indexed by *i* and *j*. This grid had a spacing of 0.025 mm; X ranged from −20 to 20 mm and Z ranged from 0 to 80 mm. The final step involved minimising the squared differences between *t*_*m*_^(*k*)^ and *t*_*s*_^(*k*)^:

1 $$(\tilde{x},\tilde{z})=\begin{array}{c}{\rm{argmin}}\\ ({x}_{i},\,{z}_{j})\end{array}\,\{\frac{{\sum }_{k=1}^{4}\,{\{[{t}_{m}^{(k)}-{t}_{s}^{(k)}({x}_{i},{z}_{j})]{w}^{(k)}\}}^{2}}{{\sum }_{k=1}^{4}{[{w}^{(k)}]}^{2}}\}$$

where the signal amplitudes at the coordinates (*h* ^(*k*)^, *v* ^(*k*)^) were used as weighting factors, *w* ^(*k*)^. The *w* ^(*k*)^ values ensured that tracking images with higher signal amplitudes contributed more prominently to the estimates of $\tilde{x}$ and $\tilde{z}$ than others. Analysis of the signals was performed in Matlab (Natick, New Hampshire, USA).

### Tracking accuracy: measurements in water

Tracking accuracy was evaluated with the US imaging/tracking probe and needle in deionised water. The needle tip position was held constant with a mechanical mount; the probe position was varied relative to that of the needle tip with a 3-axis translation stage (nominal location accuracy ±4 µm; MTS50/M-Z8, Thorlabs, UK). To evaluate accuracy in the out-of-plane dimension (X), the X coordinate of the needle tip (measured relative to the probe) was varied from 0 to 20 mm in steps of 1 mm. These variations were performed at three depths (Z = 20, 40 and 60 mm), with the needle tip always laterally centred with respect to the probe. Similar variations were performed to evaluate accuracy in the lateral dimension (Y), at the same three depths (Z = 20, 40 and 60 mm), and with the needle tip always in the imaging plane (X = 0). For accuracy in the depth (Z) dimension, the Z coordinate of the needle tip was varied from 20 to 60 mm in steps of 1 mm, with the needle tip at three out-of-plane locations (X = 0, 10 and 20 mm) and always centred laterally with respect to the probe. At each probe position, FOH sensor data were acquired for needle tip tracking. The spatial coordinate of each needle tip position in the translation dimension was compared with a corresponding reference position obtained from the translation stage. The relative tracking accuracy was defined as the Euclidean distance between these two quantities.

### Tracking accuracy: simulations with speed of sound heterogeneity

To assess the effects of SoS tissue heterogeneity on the tracking accuracy, a Monte Carlo approach with virtual FOH sensors was used. Here, the effects of SoS tissue heterogeneity on the tracking accuracy were modeled by random variations of the time-of-flights obtained with these FOH sensors. For each point of a 2D grid in the X-Z coordinate space, a virtual FOH sensor was assigned, and 1000 random time-of-flight values were generated for each of the 4 tracking elements in the X-Z plane. Those *t*_*virtual*_^(*k*)^ values were generated based on the known distances between the tracking elements and the FOH sensor. The SoS values were independently chosen from a uniform random distribution with a mean value of 1540 m/s and maximum difference from the mean of *ε*, where *ε* was either 1 or 5% of the mean value. The larger value of *ε* was chosen to account for the approximate SoS range of 1450 m/s in fat to 1600 m/s in muscle^33, 34^. The 2D grid had a spacing of 5 mm; X ranged from −20 to 20 mm and Z ranged from 10 to 60 mm. The *t*_*virtual*_^(*k*)^ values were provided to the tracking algorithm described above, in which a constant SoS value of 1540 m/s was assumed, to obtain 1000 needle tip estimates. At each grid point, the tracking accuracy in the X and Z dimensions was defined as the mean absolute difference between the coordinates of the actual and estimated needle tip positions in those dimensions. The absolute tracking accuracy was defined as Euclidean distance between these two positions.

### *In vivo* experiments

All procedures on animals were conducted in accordance with UK Home Office regulations and the Guidance for the Operation of Animals (Scientific Procedures) Act (1986). Swine were housed in accordance with UK Home Office guidelines relating to animal welfare, and the work was conducted under Project License PPL 70/7765. Swine experiments were conducted at the Northwick Park Institute for Medical Research (London, UK). Sheep experiments were conducted at the Royal Veterinary College (London, UK) under Project License 70/7408. Ethics approval was provided by the Animal Welfare Ethics Review Boards of the Royal Veterinary College and University College London, United Kingdom.

The swine was placed under terminal anaesthesia, maintained with isoflurane, and monitored continuously. For insertions toward the spine, the swine was placed in the prone position. Under X-ray fluoroscopic guidance, the needle was inserted out-of-plane at an angle of 33 degrees relative to the surface, toward the epidural space. The needle was manually withdrawn, with a pause in the withdrawal at 6 positions. For each of these positions, 3D tracking was performed.

A time mated pregnant sheep was placed under general anaesthesia and monitored continuously. Gestational age was confirmed using US. Under US guidance, an out-of-plane needle insertion into the gestation sac within the uterine cavity was performed, with an insertion angle of 42 degrees relative to the surface. The needle was withdrawn without pauses, and US tracking was performed concurrently.

### Signal-to-Noise Ratio (SNR) analysis

The SNR was calculated for each tracking image and for each needle tip position. It was defined as SNR = S/σ, where S and σ are the maximum signal amplitude and the standard deviation of image amplitudes in an empirically-chosen rectangular noise region. This region was centred at Z = 10 mm and Y = 9.5 mm with widths of 20 mm and 19 mm, respectively (the latter corresponded to 16 transducer elements from a side array). The SNR at each needle tip position was defined as the mean of the SNRs calculated from the 4 tracking images.
